# Supplementary material for: Autonomous and informed decision-making: The case of colorectal cancer screening
Source: PLoS One. 2020 May 29;15(5):e0233308. doi: 10.1371/journal.pone.0233308 (PMC7259584; doi:10.1371/journal.pone.0233308)
Supplement: S1 File — (PDF) [file pone.0233308.s001.pdf]

Interview guide

The interview guide was developed within our research team. We discussed the main questions relevant to ask in light of the objective of our study, the order of the questions, possible follow-up questions and the wording of the questions. We first conducted two pilot-interviews, after which we made a few small adjustments in the order and wording of the main questions being asked. The headings used in the interview guide are solely for structural purposes; they were not presented or communicated as such to the interviewees.

We conducted semi-structured interviews using open questions. Depending on the flow of the conversation, the order of questions could vary as well as the necessity of asking follow-up questions.

A. <u>Dutch version (original language)</u>

I. Introductie

- Korte uitleg geven over interview proces
- Lezen informatiebrief en tekenen toestemmingsformulier
- Met toestemming van de interviewee, geluidsopname starten

II. Inleidende vragen

Als nog niet uitgenodigd voor het bevolkingsonderzoek darmkanker:
Als het goed is, heeft u gehoord over het bevolkingsonderzoek darmkanker en weet u dat u door uw leeftijd onder de groep mensen valt die zal worden uitgenodigd voor dit bevolkingsonderzoek.

1) Kunt u mij vertellen waar u over het bevolkingsonderzoek darmkanker gehoord heeft?
2) Heeft u een idee wanneer u ongeveer zal worden uitgenodigd voor het bevolkingsonderzoek darmkanker?

Als wel al uitgenodigd voor het bevolkingsonderzoek darmkanker:
Als het goed is, heeft u gehoord over het bevolkingsonderzoek darmkanker en bent u er al voor uitgenodigd.

1) Kunt u mij vertellen wanneer ongeveer u uitgenodigd bent voor het bevolkingsonderzoek darmkanker?
2) Heeft u, naast via de uitnodiging, ergens anders over het bevolkingsonderzoek darmkanker gehoord? Waar?

III. Aanbod bevolkingsonderzoek

3) Waarom, denkt u, wordt het bevolkingsonderzoek darmkanker aangeboden?
4) Wat vindt u ervan dat het bevolkingsonderzoek darmkanker er is?
5) Vindt u het fijn dat u voor dit bevolkingsonderzoek wordt uitgenodigd? Waarom wel/niet?
6) Vindt u het goed hoe het bevolkingsonderzoek darmkanker nu geregeld is? Waarom wel/niet?
    - Is er iets dat u anders zou willen? Wat & waarom?

IV. Is het een (vrije en eigen) beslissing

7) Denkt u dat er van u verwacht wordt dat u meedoet aan het bevolkingsonderzoek?

8) Voelt u zich vrij om wel óf niet mee te doen aan het bevolkingsonderzoek? Waarom wel/niet?
9) Vindt u dat het aan u is om te bepalen of u wel of niet meedoet aan het bevolkingsonderzoek darmkanker? Waarom wel/niet?

- Ziet u misschien ook een rol voor anderen hierbij, bijvoorbeeld voor mensen in uw omgeving of uw huisarts of de overheid? Waarom wel/niet? Op welke manier?

10) Vindt u dat het aangemoedigd moet worden om mee te doen aan het bevolkingsonderzoek darmkanker? Waarom wel/niet?

- Als ja: Aan wie is het om dit aan te moedigen, volgens u?

V. Eigen beslissing

Als nog niet uitgenodigd voor het bevolkingsonderzoek darmkanker:
U zult dus binnenkort een uitnodiging krijgen voor het bevolkingsonderzoek darmkanker.

11) Heeft u al een idee van wat u gaat doen?

I - Als ja:
Ia) Wat denkt u te gaan doen? Waarom denkt u dit te gaan doen?
Ib) Heeft u nog twijfels over wat u gaat doen? Wat voor twijfels?
Ic) Wat zou voor u een reden zijn om eventueel [niet/wel mee] te doen?
Id) Kunt u mij vertellen wat er voor u nodig was om te kunnen zeggen dat u waarschijnlijk [wel/niet mee] gaat doen?

- Eventuele vervolgvraag: Heeft u het idee dat gevoel of emotie ook mee heeft gespeeld?
    - En gewoonte?

Ie) Heeft u ook met anderen gesproken over wat u zou kunnen gaan doen? Met wie? Wat was hun mening?
If) Denkt u dat wat u gaat doet, wel of niet mee doen aan het bevolkingsonderzoek darmkanker, ook belangrijk is voor anderen (bv. gezin, familie, zorgsysteem)?

II - Als nog niet:
IIa) Wat zijn punten/dingen die voor u meespelen?
IIb) Heeft u ergens twijfels over? Waarover?
IIc) Wat zou voor u een reden zijn om eventueel wel mee te doen? En wat zou voor u een reden zijn om eventueel niet mee te doen?
IId) Kunt u mij vertellen wat u denkt dat er voor u nodig is om te kunnen zeggen dat u waarschijnlijk wel OF niet mee gaat doen?

- Eventuele vervolgvraag: Denkt u dat misschien gevoel of emotie ook mee kan spelen?
    - En gewoonte?

IIe) Heeft u ook met anderen gesproken over uw wat u zou kunnen gaan doen? Met wie? Wat was hun mening?
IIf) Denkt u dat wat u gaat doen, wel of niet meedoen aan het bevolkingsonderzoek darmkanker ook belangrijk is voor anderen (bv. gezin, familie, zorgsysteem)?

Als wel al uitgenodigd voor het bevolkingsonderzoek darmkanker:
U heeft dus eerder een uitnodiging gekregen voor het bevolkingsonderzoek darmkanker.

11 - III) Heeft u wel of niet meegedaan?
III:
IIIa) Waarom heeft u [wel/niet mee] gedaan?
IIIb) Had u twijfels over wat u ging doen? Wat voor twijfels?
IIIc) Wat zou voor u een reden zijn om eventueel [niet/wel mee] te doen?

IIId) Kunt u mij vertellen wat er voor u nodig was om te kunnen zeggen dat u [wel/niet mee] ging doen?

- Eventuele vervolgvraag: Heeft u het idee dat gevoel of emotie ook mee heeft gespeeld?
    - En gewoonte?

IIIe) Heeft u ook met anderen gesproken over wat u kon gaan doen? Met wie? Wat was hun mening?
IIIf) Denkt u dat wat u doet, het wel of niet mee doen aan het bevolkingsonderzoek darmkanker, ook belangrijk is voor anderen (bv. gezin, familie, zorgsysteem)?

VI. Persoonlijke doelen/waarden rondom gezondheid/screenen

De volgende vraag kan lastig zijn om te beantwoorden, maar ik wil u toch vragen proberen er een antwoord op te geven. We hebben het net gehad over of u wel of niet aan het bevolkingsonderzoek darmkanker mee heeft gedaan/gaat doen.

12) Hoe past het (uiteindelijk) wel of niet meedoen aan het bevolkingsonderzoek darmkanker bij wat u belangrijk vindt in het leven?

- Eventuele toelichting/vervolgvraag: Met andere woorden, wat is voor u belangrijk in het leven en hoe wilt u uw leven leven?

13) Kunt u mij vertellen op wat voor manier gezondheid een plek inneemt in uw leven?
14) Bent u zelf veel bezig met uw gezondheid?
15) Ziet u een rol voor de overheid als het gaat om uw gezondheid? (En die van andere mensen.) Waarom wel/niet? Op welke manier?

Voor alleen vrouwen:
16a) In Nederland bestaan ook andere bevolkingsonderzoeken naar kanker, doet u daaraan mee? Waarom wel/niet?

Voor alleen mannen:
16b) Als er in Nederland andere bevolkingsonderzoeken naar kanker zouden zijn voor mannen, zou u daar dan aan meedoen? Waarom wel/niet?

VII. Wanneer een goede beslissing

Ik wil nog even terug komen op uw uitnodiging voor het bevolkingsonderzoek darmkanker.

17) Is het voor u belangrijk dat u een goede beslissing neemt over of u wel of niet meedoet aan het bevolkingsonderzoek darmkanker? Waarom wel/niet?
18) Dit kan een lastige vraag zijn, maar kunt u mij vertellen wanneer u vindt dat u een goede beslissing heeft genomen over wel of niet meedoen aan het bevolkingsonderzoek darmkanker? Waarom?

- Eventuele toelichting/vervolgvraag: Wat houdt een goede beslissing voor u in? Wat is daarvoor nodig of belangrijk? Alles wat er in u opkomt, is goed.
    - Vervolgvraag: Is er nog iets anders wat voor u van belang is?
- Eventuele vervolgvraag: Als we het hebben over *hoe* u de beslissing neemt, wanneer vindt u dat dat voor u op een goede manier gebeurt?

19) Kunt u mij ook vertellen wanneer u vindt dat u géén goede beslissing heeft genomen?
20) Wat is voor u het belangrijkste verschil tussen het nemen van een goede beslissing en het nemen van geen goede beslissing?
21) Kunt u zich voorstellen dat er dingen zijn die het nemen van een goede beslissing voor u lastiger maken? Wat & waarom?
22) Kunt u zich voorstellen dat er dingen zijn die het nemen van een goede beslissing voor u gemakkelijker maken? Wat & waarom?

23) Geldt wat we nu besproken hebben over het nemen van een goede beslissing alleen voor een beslissing over het bevolkingsonderzoek darmkanker, of ook voor andere beslissingen over gezondheid?

- Eventuele vervolgvraag: Wat is er eventueel anders? Waarom?

24) Denkt u dat anderen u nog zouden kunnen helpen bij het nemen van een goede beslissing? Bijvoorbeeld uw huisarts, de overheid of mensen in uw omgeving. Hoe?

VIII. Afsluitende vraag

25) Als laatste vraag, wat is voor u het belangrijkste als het gaat om de beslissing om wel of niet aan het bevolkingsonderzoek darmkanker mee te doen? Waarom?

IX. Afsluiting

- Afronden
- Vragen of er nog vragen zijn en of men nog iets wil toevoegen
- Bedanken voor deelname

B. English version

I. Introduction

- Give a short explanation of the interviewing process
- Reading the information letter and signing the informed consent form
- With permission of the interviewee, start audio recording

II. Introductory questions

If not yet invited for the CRC screening programme:
I believe you have heard about the CRC screening programme and are aware that because of your age you are eligible to be invited for it.

1) Could you tell me where you have heard about the CRC screening programme?
2) Do you have any idea when, approximately, you will be invited for the CRC screening programme?

If invited for the CRC screening programme:
I believe you have heard about the CRC screening programme and have received an invitation for it.

1) Could you tell me when, approximately, you were invited for the CRC screening programme?
2) Besides through the invitation, have you heard anywhere else about the CRC screening programme? Where?

III. Offer CRC screening programme

3) Why do you think that the CRC screening programme is being offered?
4) What do you think of the existence of the CRC screening programme?
5) Do you like to be invited for the CRC screening programme? Why/why not?
6) Do you agree with how the CRC screening programme is being organised? Why/why not?

- Is there something you would like to see different? What & why?

IV. Is it a decision (and a free and personal one)

7) Do you think it's expected of you to participate in the CRC screening programme?
8) Do you feel free to either participate or not participate in the CRC screening programme? Why/why not?
9) Do you believe it's up to you to determine if you should or should not participate in the CRC screening programme? Why/why not?

- Do you potentially see a role for others in this decision, for example people close to you or your general practitioner or the government? Why/why not? In what way?

10) Do you believe participating in the CRC screening programme should be encouraged? Why/why not?

- <u>If so:</u> Who do you believe should give this encouragement?

V. Own decision

If not yet invited for the CRC screening programme:
Soon you will be invited for the CRC screening programme.

11) Do you have any idea yet about what you are going to do?

I – If yes:
Ia) What do you think you are going to do? Why do you think this is what you are going to do?
Ib) Do you have any doubts about what you are going to do? What kind of doubts?
Ic) What would be a reason for you to possibly [participate/not participate] in the CRC screening programme?
Id) Could you tell me what was necessary for you to be able to say that you are probably going to [participate/not participate]?

- Possible follow-up question: Do you think that feeling or emotion played any part in this?
    - And habit?

Ie) Did you speak with other people about what you could do? With whom? What was their opinion?
If) Do you think that what you are going to do, participating or not participating in the CRC screening programme, is also relevant for others (e.g. family, care system)?

II – If not:
IIa) What are the aspects/things that are relevant for you in this?
IIb) Do you have any doubts? About what?
IIc) What would be a reason for you to possibly participate in the CRC screening programme? And what would be a reason for you to possibly not participate in it?
IId) Could you tell me what you think would be necessary for you to be able to say that you would probably participate OR not participate in the CRC screening programme?

- Possible follow-up question: Do you think that feeling or emotion could play any part in this?
    - And habit?

IIe) Did you speak with other people about what you could do? With whom? What was their opinion?
IIf) Do you think that what you are going to do, participating or not participating in the CRC screening programme, is also relevant for others (e.g. family, care system)?

If invited for the CRC screening programme:
You have previously been invited for the CRC Screening programme.

11 - III) Did you participate in the CRC screening programme or did you not participate?
III:
IIIa) Why did you [participate/not participate]?

IIIb) Did you have any doubts about what you were going to do? What kind of doubts?
IIIc) What would be a reason for you to possibly [participate/not participate] in the CRC screening programme?
IIId) Could you tell me what was necessary for you to be able to say that you were going to [participate/not participate]?
- Possible follow-up question: Do you think that feeling or emotion played any part in this?
    - And habit?

IIIe) Did you speak with other people about what you could do? With whom? What was their opinion?
IIIf) Do you think that what you are going to do, participating or not participating in the CRC screening programme, is also relevant for others (e.g. family, care system)?

VI. Personal goals/values concerning health/screening

This next question might be difficult to answer, but I would still like to ask you to try to answer it. We have just talked about whether you have participated or not participated/would participate or not participate in the CRC screening programme.

12) How does (eventually) participating or not participating in the CRC screening programme suit with what you find important in life?
- Possible clarification/follow-up question: In other words, what do you find important in life and how do you want to live your life?

13) Could you tell me how health plays a role in your life?
14) Are you engaged much with your health?
15) Do you see a role for the government concerning your health? (And that of other people.) Why/why not? In what way?

Only for women:
16a) In the Netherlands, other cancer screening programmes also exist. Do you participate in those? Why/why not?

Only for men:
16b) If, in the Netherlands, other cancer screening programmes for men would also exist, would you participate in them? Why/why not?

VII. When a good decision

I want to talk to you a bit more about your invitation for the CRC screening programme.

17) Do you find it important that you make a good decision about participating or not participating in the CRC screening programme?
18) This might be a difficult question, but could you tell me when you believe you have made a good decision about participating or not participating in the CRC screening programme? Why?
- Possible clarification/follow-up question: What does a good decision entail for you? What is needed or important to make a good decision? Anything that comes to mind, is fine.
    - Follow-up question: Is there anything else that is of importance to you?
- Possible follow-up question: When talking about *how* you are making your decision, when do you believe this is done in a good manner for you?

19) Could you also tell me when you believe you have not made a good decision?
20) What is for you the most important difference between making a good decision and not making a good decision?
21) Do you think there might be something that could make making a good decision more difficult for you? What & why?

22) Do you think there might be something that could make making a good decision easier for you? What & why?

23) We have talked about making a good decision. Does this only apply to a decision about participation in the CRC screening programme, or also to other health-related decisions?

- Possible follow-up question: What might be different? Why?

24) Do you think that others maybe could help you with making a good decision? For example, your general practitioner, the government or people close to you? How?

VIII. Closing question

25) Final question, concerning the decision about participating or not participating in the CRC screening programme, what is most important for you? Why?

IX. Closing

- Finishing up
- Asking about remaining questions or remarks
- Thanking for participation
