# Supplementary material for: Autonomous and informed decision-making: The case of colorectal cancer screening
Source: PLoS One. 2020 May 29;15(5):e0233308. doi: 10.1371/journal.pone.0233308 (PMC7259584; doi:10.1371/journal.pone.0233308)
Supplement: Attachment — Submitted filename: Response to Reviewers.docx [file pone.0233308.s002.docx]

**Response to editor comments**

Journal Requirements:

When submitting your revision, we need you to address these additional requirements:

**1. Comment 1:**

Please ensure that your manuscript meets PLOS ONE's style requirements, including those for file naming. The PLOS ONE style templates can be found at <http://www.plosone.org/attachments/PLOSOne_formatting_sample_main_body.pdf> and [http://www.plosone.org/attachments/PLOSOne_formatting_sample_title_authors_affiliations.pdf](https://eur04.safelinks.protection.outlook.com/?url=http%3A%2F%2Fwww.plosone.org%2Fattachments%2FPLOSOne_formatting_sample_title_authors_affiliations.pdf&data=02%7C01%7Cl.douma%40amsterdamumc.nl%7C5e81a6c8f46d47de1f9908d7aa4e2f3d%7C68dfab1a11bb4cc6beb528d756984fb6%7C0%7C1%7C637165125105493371&sdata=JiDgiy22K1B98TKNVC%2FfqJ1I1brTSpxQ05mEGCuVGjw%3D&reserved=0)

**Our response:**

We have adjusted our manuscript slightly to meet your journal’s style requirements.

**2. Comment 2:**

Please include additional information regarding the interview guide used in the study and ensure that you have provided sufficient details that others could replicate the analyses. For instance, if you developed a guide as part of this study and it is not under a copyright more restrictive than CC-BY, please include a copy, in both the original language and English, as Supporting Information. In addition, please include further details concerning the development or pre-testing of this guide.

**Our response:**

We have added the interview guide as Supporting Information and added details concerning the development of the guide in the method section.

***Revision (Methods, page 12, lines 281-285):***

See the *Supporting Information* for the interview guide (S1 File). The interview guide was developed within our research team. We discussed the main questions relevant to ask in light of the objective of our study, the order of the questions, possible follow-up questions and the wording of the questions. We first conducted two pilot-interviews, after which we made a few small adjustments in the order and wording of the main questions being asked.

***Revision (Supporting Information):***

Supporting Information file (S1) was added.

**3. Comment 3:**

We note that you have indicated that data from this study are available upon request. PLOS only allows data to be available upon request if there are legal or ethical restrictions on sharing data publicly. For information on unacceptable data access restrictions, please see <http://journals.plos.org/plosone/s/data-availability#loc-unacceptable-data-access-restrictions>.

In your revised cover letter, please address the following prompts:

a) If there are ethical or legal restrictions on sharing a de-identified data set, please explain them in detail (e.g., data contain potentially identifying or sensitive patient information) and who has imposed them (e.g., an ethics committee). Please also provide contact information for a data access committee, ethics committee, or other institutional body to which data requests may be sent.

b) If there are no restrictions, please upload the minimal anonymized data set necessary to replicate your study findings as either Supporting Information files or to a stable, public repository and provide us with the relevant URLs, DOIs, or accession numbers. Please see <http://www.bmj.com/content/340/bmj.c181.long> for guidelines on how to de-identify and prepare clinical data for publication. For a list of acceptable repositories, please see <http://journals.plos.org/plosone/s/data-availability#loc-recommended-repositories>.

We will update your Data Availability statement on your behalf to reflect the information you provide.

**Our response:**

We have updated our Data Availabilty statement.

***Revision (Availability of data and materials, pages 34-35, lines 653-659):***

The data supporting the conclusions of this article are included within the article. The full data set cannot be shared publicly as participants did not give consent for their transcripts to be shared in the public domain. The transcripts contain identifying items, which are therefore sensitive to privacy issues. Participants consented into the study with the understanding that only anonymised quotations would be publicly available. Anonymized data can be made available to qualified researchers by request to the medical ethical committee of the VU University Medical Center, who can be contacted at [metc@vumc.nl](mailto:metc@vumc.nl).

**Response to reviewer comments**

A. **Reviewer 1**
1. **Comment 1:**

The authors are presenting the results of a survey of subjects eligible for CRC screening, who have been interviewed, using in-depth interviews, about their experience of decision making within the Dutch CRC screening program.

The questionnaire was designed to assess the weight of the different components of an autonomous decision, referring to a comprehensive conceptual framework, considering the different components involved in the definition of individual’s autonomy.

The adoption of such framework allows the authors to analyze factors influencing subjects decision to participate in screening, going beyond the usual concept of informed decision making, identifying issues related to social norms as well as to individual’s values and experiences which can play a role in the decision making process, above and beyond the level of knowledge about screening and CRC.

This represents therefore a valuable contribution to the debate focused on the need to promote informed decision making in screening.

The paper is however quite long. Although the description of the framework is certainly useful for the readers, the authors, when presenting the results, might focus more on those aspects which can specifically be derived using a qualitative approach, while other aspects, such as, for example, the evaluation of the information provided by the official brochure (several studies have already provided data about the perceived quality of screening leaflets and the need for information), might be shortened.

**Our response:**

Thank you to the reviewer for their comment. As ‘informed’ and ‘autonomous’ are the key components of the concept of informed decision-making (IDM), we believe it is important to examine and discuss both aspects in relation to each other. Additionally, in our study, we explicitly asked participants what information they received/were aware of, and how they used this information in their actual decision-making *process*. This is somewhat different from assessing general information needs, which has been a common approach in previous studies. However, we agree that our manuscript is quite long and any suggestion to make it shorter is more than welcome. Thus, we have taken another look at our result section and made a few changes (i.e. deleted less relevant information) to shorten it (see the revision below).

***Revision (Results, page 15, lines 327-340):***

***3.2.1. Role of information and the official brochure***

Participants and non-participants of the CRC screening programme indicated wanting to know about the basics of more or less the same aspects regarding CRC screening (e.g. the test procedure, the screening purpose, and the possible general benefits, harms and downsides of screening) (Quote 1, Table 2). Most CRC screening participants and non-participants mentioned they heard or were informed about generally all of the aspects they wanted to know about (we did not systematically assess their knowledge). However, some CRC screening participants and non-participants mentioned not to have heard about any downsides or risks associated with CRC screening and were unclear about the target population and procedure of the CRC screening programme. Several CRC screening participants and non-participants mentioned that information was mostly needed regarding what to expect once you have decided to participate in CRC screening. Most CRC screening participants and non-participants had read or browsed through the official brochure accompanying the CRC screening invitation, or intended to do so after receiving the invitation (Quote 2, Table 2). Most already had a clear idea about participating before actually receiving the CRC screening invitation and official brochure (Quote 3, Table 2).

2. **Comment 2:**

The number of subjects interviewed is really small, and as indicated also by the authors, subjects in the lowest educational level are severely under-represented in the group of non-participants.

I am not an expert in the field of qualitative research and I can imagine that the process of fixing, conducting and analysing these interviews is resource demanding. So the choice to stop the interviews when no new themes emerged, might be justified both from a methodological point of view as well as from a feasibility perspective. However, I would think that this choice may allow to indicate which are the themes reported by the different subgroups, but it may not allow to conclude whether a specific theme is associated with different sub-groups (participants or non-participants, for example), or which is the its relative weight as compared to other factors. I would consider to address this issue in the discussion.

**Our response:**

We agree with the reviewer that we should be careful with making conclusive statements that are not supported by the qualitative data and that this issue should be addressed. The purpose of our qualitative study is exploring the relevant themes that are involved in the decision-making process, among both CRC screening participants and non-participants. Certain themes were mentioned clearly more often among non-participants than among participants, which we believe is worth mentioning. Following the reviewer’s comment, we have taken another look at our discussion section regarding how to report certain findings and made a few changes. Additionally, in the introduction section, we clarified the exploring nature of our study.

***Revision (Abstract, page 2, lines 43-44):***

This appeared to be the case to a lesser extent for CRC screening participants.

***Revision (Introduction, page 9, lines 225-228):***

Therefore, using in-depth interviews, , which provides us the opportunity to delve in deeper into the process of decision-making, the aim of our study was to explore how the concepts of autonomous and informed decision-making relate to: …

***Revision (Discussion, page 27, lines 468-469) :***

However, in our small sample, this view appeared to be more evident among CRC screening non-participants than among participants.

***Revision (Discussion, page 27, lines 475-477):***

However, the social pressure expressed by CRC screening non-participants in our sample to participate in the CRC screening programme may be affecting the freedom of choice of especially those considering not to participate in CRC screening.

***Revision (Discussion, page 28, lines 494-498):***

To foster freedom of choice and making informed decisions concerning CRC screening participation, the possibility of a previously established social norm should be acknowledged and addressed in public communication regarding CRC screening. It should also be realised that information about CRC screening is likely to be processed within the frame of CRC screening being beneficial.

***Revision (Discussion, page 29, lines 509-512):***

However, in our sample, especially CRC screening non-participants appeared to attribute an important role to information and deliberation when deciding about CRC screening participation, similar to the view of cancer-screening experts with their focus on informed decision-making (7-9).

***Revision (Discussion, page 29, lines 519-521):***

That being said, we should realise that, in general, our sample is too small to draw any conclusions from. The proposed possibility would be, however, interesting to further explore in future research.

***Revision (Discussion, page 30, lines 548-551):***

Considering our small study sample, we should be careful drawing conclusions, but perhaps our findings indicate a difference in the values and perspective between people deciding to participate in CRC screening and people deciding not to participate.

***Revision (Conclusion, page 33, lines 626-628):***

However, in particular the existence of a social norm may be affecting a true autonomous decision-making process.

3. **Comment 3:**

The final suggestion to consider the option to reconsider the choice of adopting an active invitation strategy, based on the reported feeling of social pressure to participate by non- participants. The unbalanced distribution of the strata of educational level and the limited size do not allow to reach sound conclusion about the weight of such factor. It definitely plays a role (as also reported from other studies) and the authors are also describing the potential influence of such social norm on the way people are processing information about screening. On the other hand, the invitation does not only represent a strategy aimed to overcome practical barrier. Strong evidence is indeed supporting the hypothesis that an active invitation strategy is associated with a reduction of disparities in access to screening (and subsequent caner care). Self-initiated screening is more often a decision of better educated people, showing higher health awareness and the necessary competence, knowledge and literacy skills to consider taking screening. Avoiding widening the SES gap while promoting informed choice represents a challenge which should not be underevaluated.

**Our response:**

We agree with the reviewer that the positive effects of an active invitation strategy should not be underevaluated. The purpose of this segment in the discussion section is to spark a bit of debate, to ensure that all possible benefits and downsides are considered when ‘people in charge’ are deciding about their invitation strategy. Different interests, obstacles and starting positions can be involved and all should be considered to ensure an invitation strategy that is best suited for the particular context at that time. Thus, we believe that at least a discussion about all possible effects should precede making the decision about what kind of invitation strategy to use. Following the reviewer’s comment, we have made a few changes to the discussion section.

***Revision (Discussion, pages 31-32, lines 569-584):***

Additionally, it might be worth considering, or at least starting a debate about, revising or adjusting the active screening invitation strategy that is currently part of the Dutch cancer screening programmes (as well as being used in a number of other countries (29, 30)). An active invitation strategy can have significant positive effects, mainly eliminating certain practical barriers for participating in CRC screening and more equal access to CRC screening (e.g. improved participation rates of people from lower SES groups) (52, 63, 64). However, using an active invitation strategy may also have an undesired effect. It might be contributing to the perpetuation of the existing social norm regarding participation (40-42) as well as to people possibly not considering what CRC screening participation actually entails (65). Perhaps the existence of a certain barrier, such as requesting the stool test themselves, is a desired stimulant for people to actively think about their screening decision and consider the possible implications of participating and not participating. Thus, providers of CRC screening are confronted with the complex matter of, on the one hand, enhancing equal access to screening and, on the other hand, promoting informed decision-making and informed participation/non-participation. Therefore, providers should adequately discuss both objectives and all pros and cons associated with a chosen invitation strategy.

B. **Reviewer 2**
1. **Comment 1:**

In the manuscript the authors are presenting very interesting and still understudied field of autonomous and informed decision-making in cancer screening – precisely in colorectal cancer screening. Therefore, I find the study results of high importance for health authorities and personnel that are involved in the cancer screening and its organization.

Certain parts of the manuscript are difficult to understand (for instance lines 27, 28-29, 214), I would advise that a native speaker revises the text.

**Our response:**

The manuscript had already been proofread by a native (UK) English speaker before submitting the manuscript. However, following the reviewer’s comment, we asked the native speaker to proofread the manuscript again.

2. **Comment 2:**

I would advise also to consider using the word “harms” instead of “downsides”.

**Our response:**

We understand where the reviewer is coming from and have previously discussed within our team which term would be best to use (harms or downsides?). In general, we believe that ‘harms’ is a less inclusive term, mainly accentuating clearly harmful consequences, while ‘downsides’ covers a broader range of possible negative consequences. Additionally, using the broader term ‘downsides’ leaves room for the different weights that different people might attach to the different negative consequences that could occur. Therefore, we initially decided to use the term ‘downsides’ and not ‘harms’. However, following the reviewer’s comment, we now changed it to ‘harms and downsides’ to include all possible interpretations.

***Revision (throughout the manuscript):***

Throughout the manuscript ‘downsides’ was changed to ‘harms and downsides’. See the changes highlighted in yellow.

3. **Comment 3:**

Lines 251-256 should go into discussion part.

**Our response:**

We agree with the reviewer that the information mentioned here should also be mentioned in the discussion section, which it is. However, we believe it is also relevant to mention this information in the method section, as it was part of our selection strategy and procedure. We did make some adjustments to the wording and deleted some of the text to be more in line with it being a description of our method. The deleted parts can still be found in the discussion section *(pages 32-33, lines 610-613)*.

***Revision (Methods, page 11, lines 271-277):***

We tried to oversample low educated people as their views can be underrepresented in research. Unfortunately, they were underrepresented in our ‘starting-pool’ of possible interviewees. Additionally, despite efforts on our end to accommodate them and for diverse reasons (e.g. not picking up their phone, no time, other priorities, other unmentioned experienced barriers), they proved to be more difficult to actually schedule an interview with. Participants were all in the average risk population. We stopped conducting interviews when no new main themes were identified in the data.

C. **Reviewer 3**
1. **Comment 1:**

Rationale for study:

- Rationale for focus on autonomy in addition to informed decision is set out as a key rationale for the paper. But it is not clear if CRC screening conducted through primary care providers would not meet the shared decision-making context, and how autonomy might be supported in that context.

**Our response:**

In our study, we are addressing how informed decision-making (IDM) is being applied within the context of CRC screening. In many countries, such as the Netherlands, but also for example the UK, Australia, Italy, Sweden, and Finland, CRC screening is being offered following an IDM approach. In some regions of the USA this is also the case, although a shared decision-making (SDM) approach is also a common approach, often following the paradigm of opportunistic screening instead of organised screening. In the medical clinical area, SDM is being promoted as a way to stimulate people’s involvement and sense of autonomy. In the medical preventive area, especially regarding screening, IDM is being promoted as a way to stimulate people’s involvement and sense of autonomy. This is partly because regarding certain preventive actions, such as large-scale population-based preventive cancer screening, it is less costly. Therefore, our focus is on examining the concept of IDM, and not SDM, and whether it is corresponding with how people in reality make their CRC screening decision.

To further clarify the concept of informed decision-making, the difference with shared decision-making and the use of it in the Netherlands and other countries, we made a few changes to our introduction. Additionally, we made some changes to the structure of the introduction (i.e. we mentioned the part that discusses IDM and SDM sooner now, and we placed the text discussing CRC screening in the Netherlands under a separate paragraph *‘1.2. CRC screening in the Netherlands’*). This hopefully contributes to a better understanding of our rationale as well. In the revised manuscript, the newly added text is highlighted in yellow. The changes made to the structure are highlighted in green (only shown in the manuscript, not in the rebuttal-document).

***Revision (Introduction, page 4, lines 79-81):***

Concepts such as empowerment (3), self-management (4), shared decision-making (5), informed decision-making (6) and autonomy (7) have become an essential part of medical care and public health.

***Revision (Introduction, page 5, lines 112-114):***

Thus, making an informed decision can be the result of a shared decision-making process, but the *concept of informed decision-making* as such follows an individual decision-making process, without active involvement of a health professional.

2. **Comment 2:**

- The rationale for focusing solely on CRC screening for understanding something more generic – autonomous and informed decision-making – was not clearly laid out. Is there anything about cancer screening decisions or CRC screening decisions in particular that may affect the study results? The authors cite three articles for why they focus on CRC screening but don’t explain the rationale in the paper. They need to make a better case for why they focus on CRC screening and what the implications of that are.

- It seems that these findings have fairly narrow relevance to the case of CRC screening. Even generalizing to other types of cancer screening may be problematic given the unique nature of the screening test, follow-up of positive tests, etc.

**Our response:**

In our study, we are addressing the concept of IDM as such, and how this is being translated/executed in practice. As IDM is being promoted within the field of cancer/CRC screening, we believe it is relevant to explore how IDM is being applied within this context *(see our Introduction, page 4, lines 84-89).* We have chosen to focus on CRC screening for several reasons: 1) In the Netherlands, this was the newest cancer screening programme being introduced, which happened to be in a time-period (2014) where IDM is strongly being promoted. Thus, right from the start an IDM approach is actively being used regarding the Dutch CRC screening programme. 2) Between CRC screening and other types of cancer screening (e.g. breast and cervical) there are both similarities and differences. The differences warrant a focus on solely CRC screening, which is also not uncommon when using a qualitative design in order to explore a specific topic, while the similarities warrant a generalizability of our study results on certain shared aspects. The differences pertain, as mentioned by the reviewer, to the screening tests and follow-up tests being used. The similarities pertain to the possibility of false-positives and false-negatives, psychological distress, expected or experienced discomfort with the tests being performed, and overdiagnosis and overdetection. These are all possible harms and downsides that are present regarding all types of cancer screening. Additionally, in the Netherlands all types of cancer screening follow a similar organisation design, where people are invited by the government, receive an information brochure and no medical professional is involved in the initial decision-making process. The way the CRC screening programme is organised in the Netherlands is not unique to the Netherlands, as mentioned under comment 1. Although differences still exist between countries where there are organised screening programmes, there are also similarities in how these programmes are organised and in the screening procedures that are being used.

To clarify our rationale, we made some changes to our introduction section.

***Revision (Introduction, page 4, lines 82-84):***

… ,with many experts in the field of cancer screening currently and increasingly considering it important that people make a personal and informed decision regarding screening participation (8-10).

***Revision (Introduction, page 4, lines 90-92):***

These benefits, harms and downside are, in general, similar to the benefits, harms and downsides associated with other types of cancer screening, although of course different screening tests are being used.

***Revision (Introduction, page 5, lines 119-120):***

From the beginning, informed decision-making regarding CRC screening is emphasised as important (26-28).

***Revision (Introduction, pages 5-6, lines 120-126):***

The Dutch screening programmes are organised by the government, which is similar to a number of other countries, such as the United Kingdom (UK), Australia, Italy and Finland (29, 30), but also not the standard situation worldwide. With organised cancer screening, people are actively invited to participate in cancer screening and the uptake is monitored. However, other countries, such as Germany or the United States of America (USA), rely on opportunistic screening (31, 32), where, in essence, the responsibility lies with the individuals themselves to ask their physician to be screened, and the uptake may not be monitored.

***Revision (Discussion, page 33, lines 614-621):***

In our study, we focused on CRC screening and not also on other types of cancer screening because in the different screening programmes different screening tests are being used. However, the benefits, harms and downsides associated with CRC screening are, in general, similar to the benefits, harms and downsides associated with other types of cancer screening. Additionally, although not the standard situation worldwide, the organisation of CRC screening in the Netherlands is similar to its organisation in a number of other countries, such as the United Kingdom (UK), Australia, Italy and Finland (29, 30). Therefore, we believe our study results are relevant for other types of cancer screening and other countries.

3. **Comment 3:**

‘Good’ decision

- What is a ‘good’ decision? Decision conflict? Decision confidence? Decision satisfaction? Not clear how the authors define what is a ‘good’ decision, despite using quotations for ‘good’ throughout the paper. Although the a ‘good’ decision is the focus on the second research question, the results are not clearly presented. Were participants asked if they felt they had made a ‘good’ decision, or only to comment on what they thought a ‘good’ decision was? This needs to be clarified. If all participants indicate that they made a good decision, then they are really justifying how they made their decision – which is a different consideration.

**Our response:**

As the reviewer mentions, one of our research questions involves exploring what people themselves consider to be a ‘good’ screening decision. This because the whole concept of IDM (and SDM) is mainly based on what experts consider to be a ‘good’ decision (see in our introduction lines 84-88, and also: Ratliff et al. What is a good decision? Effective Clinical Practice. 1999;2:185-197). We realise that the possibility exists that participants are justifying their decision. We have asked participants in our study the question ‘When do you believe you have made a good decision about CRC screening participation?’. We have also asked them to answer this question in relation to health-related decisions in general. Responses pertained to both the process of decision-making and the possible outcome of their decision. This was stated in the original manuscript in the method section and the results section. However, following the reviewer’s comment, we have made some changes to the introduction and method section. In the method section we now say that we asked about these *topics* instead of referring to them as the exact main *questions* being asked. Additionally, following one of the editor’s comments (see above), we have added the interview guide as Supporting Information, which should provide a better insight into what and how the exact questions have been asked.

***Revision (Introduction, page 4, lines 84-86):***

Consequently, making an informed decision, according to the expert-defined concept of informed decision-making, is generally viewed by experts as making a ‘good’ screening decision (9-11).

***Revision (Introduction, page 8, lines 198-200):***

This in comparison to the beforementioned expert view on what constitutes a ‘good’ screening decision, namely a decision following the concept of informed decision-making (9-11).

***Revision (Introduction, pages 8-9, lines 200-202):***

Previous studies have found that people regularly failed to make an informed CRC screening decision according to the commonly used expert-definition, …

***Revision (Methods, page 12, line 289):***

Our interview guide addressed the following main topics: …

4. **Comment 4:**

- The contrast between informed and shared decision-making is briefly noted, but this deserves more attention in the paper. Is this the case because invitations to CRC screening in NL are through letter invitation and not through visit with a primary care provider?

**Our response:**

In the Netherlands, but similar to other countries, no health professional is involved in the decision-making process about screening participation. It is an individual informed decision-making process. A shared decision-making process regarding CRC screening is indeed not part of the Dutch context. See also our response and revisions under comment 1.

5. **Comment 5:**

Methodology:

- The methodology/study design is not explained. The authors note that in depth interviews will be used to collect data but no overarching study design is noted.

**Our response:**

In section 2.3 (pages 11-12) we explain how the data collection and analysis has taken place (semi-structured in-depth interviews using open questions, followed by a descriptive thematic analysis). It describes the qualitative design and methodology used. We have added a sentence in section 2.3 to make the design explicit.

***Revision (Methods, page 12, line 280):***

We used a qualitative design, using in-depth interviews.

6. **Comment 6:**

- Unclear why study relied solely on interviews. Further to above point, a case study approach of different programs would have strengthened the study considerably and allowed for more generalizable results.

- Important limitations of studying decision-making with only one programme context (NL). Why a broader study in other jursidctions wasn’t contemplated is unclear, but would have led to a much stronger study.

**Our response:**

Interviews provide the opportunity to explore a topic in more depth and to address follow-up questions. As mentioned under comment 2, we recognise that there are differences between different types of cancer screening and different countries. However, there are also many similarities (especially with the other countries that also offer organised screening programmes). We believe that the differences warrant a focus on solely CRC screening, while the similarities warrant a generalizability of our study results on certain shared aspects. Our study offers relevant insights regarding the concept of informed decision-making and its execution in the field of CRC/cancer screening, which can contribute to the - ongoing - debate on how to best support people in making a decision about CRC/cancer screening. We address both these points in the limitation section (see also under comment 2).

***Revision (Introduction, pages 9-10, lines 225-227):***

Therefore, using in-depth interviews, which provides us the opportunity to delve in deeper into the process of decision-making, the aim of our study was to explore how the concepts of autonomous and informed decision-making relate to: …

***Revision (Discussion, page 32, lines 587-589):***

Our qualitative study offers relevant insights regarding the concept of informed decision-making in the context of CRC screening and contributes to the debate on how best to support people in making a decision about CRC screening.

7. **Comment 7:**

- Used national online research panel – unclear why given the numbers (only 27 interviewees), a more traditional (and less biased – they have to sign up and they receive a 20 EUR gift card) approach to recruit participants wasn’t used.

**Our response:**

While we agree with the reviewer that using an online panel brings a certain bias with it, we believe this is fairly common practice and in our case would be the best approach. The CRC screening programme in the Netherlands was gradually being implemented between 2014 and 2019. When we conducted our study, not all birth years theoretically eligible for CRC screening were being invited yet. We needed a sample that was actually already invited for the CRC screening programme or was soon about to be invited. Using a research panel was the best approach as they know people’s birth year, which we could use as a filter. The 20 EUR gift card was provided by us, the researchers, not by the research panel. Following the reviewer’s comment, we have made a few small changes to make the advantage of using an online research panel in our study more clear.

***Revision (Methods, page 10, lines 242-243):***

To thank interviewees for their participation, we provided them with a 20-euro gift card.

***Revision (Methods, pages 10-11, lines 250-253):***

In theory, everyone between 55 and 76 years old was eligible to be invited for the CRC screening programme. However, in the Netherlands, the CRC screening programme was gradually being introduced over a period of five years (2014-2019) among different birth cohorts.

***Revision (Methods, page 11, lines 255-257):***

In the Netherlands, we do not have a publicly available database of people being invited for CRC screening. Therefore, we used a research panel as they know people’s birth year, which we could use as a filter. Thus, eventually, …

8. **Comment 8:**

- The two groups were not what I anticipated. The first group were invitees (first time). Second group were not invited. I was expecting the second group who had been invited but decided not to go forward with screening.

- Not clear enough what the sample was. What they had to answer to be eligible.

- Interviewees were asked about experience making CRC screening decision, yet some participants come from a group that had not yet been invited – so what did that entail?

- Why did the authors include those who had already made the decision and those that had not (intention group)? They suggest that this is a strength of the study, but then they combine the results for these two groups, which does not allow the distinction between these two groups to be assessed. This is an important limitation.

**Our response:**

Reading the reviewer’s comment, we realise that we have not been clear enough in describing the group of interviewees that we researched. We were interested in examining the decision-making process of people who were invited for the first time for CRC screening. This decision-making process would not only happen on the day that people receive their invitation or on the day that they actually take the screening test, but also some time before that. Thus, we recruited participants who, based on the invitation schedule, had relatively recently been invited/made their decision or who were expected to be invited/make their decision soon. Following the reviewer’s comment, we have described the process regarding eligibility for our study more clearly. As mentioned in our limitation section, we realise that asking people about their decision before having acted on it brings possible limitations with it. However, asking people about their decision afterwards also brings certain limitations with it. Therefore, we tried to include both groups as part of our sample. We found no real differences between the two groups. This is something we could also mention in the manuscript.

***Revision (Introduction, page 10, lines 228-229):***

1) How the eligible CRC screening population in actuality decide about first-time participation in the Dutch national preventive CRC screening programme; …

***Revision (Methods, pages 10-11, lines 243-250):***

For our study, we were interested in examining the decision-making process of people who were invited for the first time for CRC screening. This decision-making process would not only happen on the day that people receive their invitation or on the day that they actually take the screening test, but also some time before that. Especially since the existence of the CRC screening programme and who was eligible to be invited had received a reasonable amount of media attention. Therefore, we recruited participants who, based on their birth year, relatively recently received an invitation for CRC screening/made their decision, or who were expected to receive an invitation/make their decision soon.

***Revision (Methods, page 11, lines 261-263):***

From these respondents it was also known whether or not they had participated in the CRC screening programme or what their intention was regarding participation.

***Revision (Methods, page 12, lines 285-286):***

We asked people about their experience with making, or soon to be making, …

***Revision (Discussion, page 32, line 605):***

Between the two groups we found no real differences.

9. **Comment 9:**

- The authors make note about challenges recruiting lower education participants (but not clear why scheduling issues were not surmountable).

**Our response:**

During the planning of the interviews, we experienced that for diverse reasons people with a lower education (who were already less present in our ‘starting-pool’ of possible interviewees) were more difficult to actually schedule an interview with. We had more difficulty to plan an interview with them. We made several attempts to accommodate them (interviews could take place at their home or other preferred location, in the evening or in weekends), but even then we were unable to interview these people, even though they initially signed up for it. It is unfortunately a common issue that people from groups with a lower education or social-economic status are more difficult to include. Following the reviewer’s comment, we made a few changes to the method and discussion section.

***Revision (Methods, page 11, lines 272-275):***

Unfortunately, they were underrepresented in our ‘starting-pool’ of possible interviewees. Additionally, despite efforts on our end to accommodate them and for diverse reasons (e.g. not picking up their phone, no time, other priorities, other unmentioned experienced barriers), they proved to be more difficult to actually schedule an interview with.

***Revision (Discussion, page 32, lines 594-598):***

This group was underrepresented in our ‘starting-pool’ of possible interviewees to begin with and proved, for diverse reasons, to be more difficult to actually schedule an interview with, which affects generalizability and limits the conclusions we can draw. It is unfortunately a common issue that people from groups with a lower education or social-economic status are more difficult to include in research.

10. **Comment 10:**

- Assume quotes were translated from Dutch to English – how translation was done should be clarified in the methods.

**Our response:**

We have included it in the method section.

***Revision (Methods, page 13, lines 304-305):***

The interview guide and the quotes in Table 2 (Results) were translated from Dutch to English by the first author and proofread by a native (UK) English speaker.

11. **Comment 11:**

Results:

- The characteristics of the two groups differ in terms of sex, children and age (to varying extents). The authors need to unpack the implication of this more.

**Our response:**

The focus of our study is on CRC screening participants versus non-participants. In qualitative research, especially concerning small samples like ours, it is less common and relevant to analyse results in detail according to certain sociodemographic characteristics. We should be careful with making inferences and drawing conclusions based on the differences in the small sample of numbers presented here. The objective of a qualitative approach is to collect a range of experiences and views from a diverse set of people, as balanced as possible. Regarding the characteristics sex, age, relationship status and having children, we believe that the differences between the two groups (CRC screening participation and non-participation), considering the small sample it concerns, are not large enough to draw conclusions from. We have added a comment about this in the discussion section. We also mentioned that, because of the limited invitation schedule concerning CRC screening, we could not include people from the age-range 65-69.

***Revision (Results, legend Table 1, page 14, lines 322-323):***

- Because of the gradual introduction of the CRC screening programme in the Netherlands, people from the age category 65-69 were not invited for CRC screening during our study period (2017/2018)

***Revision (Discussion, page 32, lines 589-593):***

Our interview sample consisted of a sample of both participants and non-participants of the CRC screening programme, which was divers in sex, age, education, relationship status and having children. Considering our small sample size, the differences on sociodemographic characteristics between CRC screening participants and non-participants could not be analysed.

***Revision (Discussion, page 33, lines 601-602):***

Additionally, our study did not include people from the age-range 65-69, due to the gradual implementation of the CRC screening programme.

12. **Comment 12:**

- Regarding participants, also not clear if any of the sample would be classified as higher risk populations in terms of CRC screening (e.g., have direct family members who had colorectal cancer). The assumption is that the participants were all in the average risk population – but section 3.4.2 possibly suggests otherwise. This should be explicit.

**Our response:**

Participants were all in the average risk population. We have added this to the method section to make this explicit.

***Revision (Methods, page 11, lines 275-276):***

Participants were all in the average risk population.

13. **Comment 13:**

- I appreciated Table 2 – this was an effective way to present qualitative interview data that doesn’t negatively affect the flow of the narrative of the paper.

**Our response:**

Thank you.

14. **Comment 14:**

Summary

Overall, while an interesting study, there are many limitations that affect the generalizability of the findings. Part of this is due to the methodological approach, which is quite basic, part of this is conceptual with issues related to the rationale for the study and in particular how ‘good’ decisions were presented both conceptually and empirically. Ultimately, the results are relevant only to CRC screening decisions in the context of public programs that use a mass invitation protocol (rather than a primary care model). This is fairly narrow for an interest in informed/autonomous decision-making – and the authors need to make a much stronger case for how to position these findings in a broader context, or alternatively, make case for why the narrow CRC screening focus is justified.

**Our response:**

As this comment is a summary, we would refer to our responses on the previous comments, where we believe all the points mentioned here have been addressed. In general, the benefits, harms and downsides associated with CRC screening are similar to the benefits, harms and downsides associated with other types of cancer screening. Additionally, although not the standard situation worldwide, the organisation of CRC screening in the Netherlands is similar to its organisation in a number of other countries. Therefore, we believe our study results are relevant for other types of cancer screening and other countries.

15. **Comment 15:**

- The authors use ‘…cancer screenings…’ in a number of places. This should be corrected to ‘…types of cancer screening…’ or ‘…cancer screening programs…’.

**Our response:**

We have made the suggested change. See the highlighted text segments throughout the manuscript.

16. **Comment 16:**

- Line 122 – “This process could stimulate…”. It’s not clear whether ‘this’ refers the to sentence immediately preceding it or the sentence before that. This should be tweaked to be clear.

**Our response:**

We have made the suggested adjustment.

***Revision (Introduction, page 5, lines 109-112):***

In a shared decision-making context, a physician and patient jointly discuss and decide about the medical intervention and this could stimulate a broader perspective on the information, values and preferences involved (5, 25).

17. **Comment 17:**

- End of section 2.3 – authors identify emerging themes. This should not be presented in the data collection/analysis section – as this is a methods section. It should be in the results.

**Our response:**

We have moved the sentences concerning the emerging of themes to the beginning of the results section.

***Revision (Results, page 13, lines 308-311):***

Conducting our qualitative analysis, the following main themes emerged, which were used to structure our results: 1) Aspects related to being informed about CRC screening; 2) Aspects related to autonomous decision-making; 3) Oher aspects involved in the decision-making process; 4) A ‘good’ screening decision.

18. **Comment 18:**

- Table 1 should break down the participants by the four groups – not just two amalgamated groups.

**Our response:**

Although we understand where the reviewer is coming from, we hope that with our better description of who we included in our study (see our response under comment 8) the reviewer can understand why we believe it is justified to divide participants using our original two groups.

19. **Comment 19:**

- Line 385 – change ‘Oher’ to ‘Other’.

**Our response:**

Thank you, we revised it.

***Revision (Results, page 25, line 413):***

**3.4. Other aspects involved in the decision-making process**

20. **Comment 20:**

- Line 390 – avoid ‘—,’ together. Either use ‘—’ or ‘,’ only

**Our response:**

We have now used the (..) instead of the ‘—’.

***Revision (Results, page 25, 416-419):***

A sizeable number of CRC screening participants and non-participants indicated that certain emotions/feelings were present (such as a slight fear of cancer or colonoscopy, anxiety while waiting for the test results, anxiety when getting a negative test result, or reassurance when getting a positive test result), and that these played a role in their decision-making process (Quote 18, Table 2).

21. **Comment 21:**

- Line 394 and 395 – avoid ‘had had’.

**Our response:**

We have made the suggested adjustment.

***Revision (Results, page 25, lines 422-423):***

Most CRC screening participants and non-participants knew other people close to them who had cancer in the past, and a number of them had cancer in the past themselves.
